# MicroRNAs as multifaceted regulators and therapeutic targets in ulcerative colitis

**DOI:** 10.3389/fimmu.2025.1697059

**Published:** 2025-12-18

**Authors:** Xuerui Wang, Nan Jiang, Weixiang Yin, Xiaofeng Cui

**Affiliations:** 1 Department of Gastrointestinal Colorectal and Anal Surgery, China-Japan Union Hospital of Jilin University, Changchun, Jilin, China; 2 Department of Clinical Laboratory, The Second Hospital of Jilin University, Changchun, Jilin, China; 3 Department of Cardiovascular, The Second Hospital of Jilin University, Changchun, Jilin, China; 4 Department of General Surgery, Affiliated Hospital of Yangzhou University, Yangzhou, Jiangsu, China

**Keywords:** biomarkers, gut microbiota, immune homeostasis, microRNAs, miRNA-targeted therapeutics, ulcerative colitis

## Abstract

Ulcerative colitis (UC) is a chronic inflammatory bowel disease characterized by mucosal barrier disruption, immune dysregulation, and gut microbiota imbalance. MicroRNAs (miRNAs), small non-coding regulators of gene expression, have emerged as pivotal modulators of UC pathogenesis. By orchestrating epithelial cell apoptosis, tight junction integrity, and mucin secretion, miRNAs such as miR-223, miR-151-5p, and miR-429 contribute to barrier dysfunction. Additionally, miRNAs shape the innate and adaptive immune responses by influencing macrophage polarization, dendritic cell maturation, and T cell subset differentiation, including Th17/Treg and Th1/Th2 balance. Specific miRNAs further modulate gut microbial composition and host-microbe interactions. Clinically, circulating miRNAs serve as promising non-invasive biomarkers for disease diagnosis, activity monitoring, and therapeutic response prediction. Therapeutically, miRNA mimics and inhibitors have shown efficacy in early-phase clinical trials, offering a novel strategy beyond current biologics. This review summarizes recent mechanistic insights and translational advances, underscoring the multifaceted roles of miRNAs in UC and their potential to inform precision diagnostics and targeted therapies.

## 1 Introduction

Ulcerative colitis (UC) is a chronic immune-mediated disorder characterized pathologically by diffuse mucosal inflammation and clinically by abdominal pain, diarrhea, passage of mucus- and pus-laden bloody stools, and tenesmus (1). The incidence and prevalence of UC had risen in most Western nations as well as in newly industrialized countries (2), rendering it a substantial global healthcare burden (3). Consequently, there is an urgent clinical need for more effective diagnostic, monitoring, and therapeutic strategies for UC. MicroRNAs (miRNAs) are endogenous, non-coding, single-stranded RNAs of 19–28 nucleotides that participate in diverse biological processes, including cell growth, differentiation, proliferation, and apoptosis, and are widely recognized as pivotal post-transcriptional regulators of gene expression (3–5). Increasing evidence has revealed a close association between miRNAs and UC pathogenesis. miRNAs can directly or indirectly modulate key pathogenic regulators and signaling cascades implicated in UC onset and progression, such as retinoic acid-related orphan receptor C (RORC), forkhead box transcription factor O1 (FoxO1), and signal transducer and activator of transcription 6 (STAT6), as well as the IL-6/STAT3, MAPK, and TLR4/MyD88/NF-κB signaling pathways (6–11). These regulatory networks influence the integrity of the intestinal mucosal barrier, the composition of the gut microbiota, and immune homeostasis, thereby exerting critical roles in UC pathophysiology.

Although conventional pharmacologic interventions such as glucocorticoids remain central to UC management, biologics have emerged as effective therapeutic modalities for UC (12). However, long-term administration is usually complicated by the development of drug tolerance and a spectrum of adverse effects (13). Therapeutic strategies based on miRNA modulation represent a promising alternative to biologics. Nevertheless, their clinical translation faces formidable challenges, including rapid degradation in the circulation, low cellular uptake, and poor systemic delivery specificity (14). To date, apart from ABX464, which is currently undergoing phase II clinical evaluation, some other miRNA-targeted therapeutics (miR-29 mimics) have been explored in preclinical studies (15–18). This review synthesizes recent advances on the mechanistic relevance of miRNAs in UC pathogenesis, their potential utility as biomarkers for UC diagnosis and prognosis, and emerging miRNA-targeted therapeutics for UC, providing a conceptual and mechanistic framework to inform future diagnostic innovation and targeted therapy development for UC.

## 2 Mechanisms of miRNAs in UC progression

Mucosal barrier dysfunction, microbial dysbiosis, and immune homeostasis imbalance constitute the core pathogenic mechanisms of UC (19). Multiple studies have reported that miRNAs serve as critical regulators of the intestinal mucosal barrier, gut microbiota, and both innate and adaptive immunity, acting either directly or indirectly on colonic epithelial cells, junctional proteins, probiotic and opportunistic pathogenic bacteria, immune cell proliferation and differentiation, and inflammatory mediators.

### 2.1 miRNAs regulate UC via modulation of the intestinal mucosal barrier

The intestinal mucosal barrier serves as a critical innate defense mechanism that restricts the translocation of bacterial antigens and toxins, thereby preserving intestinal homeostasis (20). Emerging evidence underscores the role of microRNAs as pivotal regulators of epithelial barrier integrity in UC. Excessive apoptosis of intestinal epithelial cells (IECs) disrupts the proliferation–apoptosis equilibrium, diminishes the mucus layer, and enhances mucosal permeability, collectively exacerbating disease progression. Consequently, suppressing IEC apoptosis and facilitating mucosal repair represent central therapeutic objectives. Several miRNAs, including miR-330-3p, miR-223, and miR-452-5p, augment IEC apoptosis through distinct molecular pathways: miR-330-3p downregulates X-box binding protein 1 (XBP-1), a transcription factor induced by endoplasmic reticulum stress that modulates autophagy and apoptosis (21); miR-223 influences the PI3K/AKT/mTOR axis in IECs, thereby promoting apoptosis and impeding mucosal healing during inflammation (22); and miR-452-5p targets the anti-apoptotic protein Mcl-1, altering IEC responsiveness in murine models of inflammatory bowel disease (23). Beyond apoptosis regulation, miRNAs impair the mucosal mechanical barrier, a central element in UC pathogenesis. miR-151-5p upregulation increases caspase-3 and claudin-2 while reducing claudin-1 and occludin; exogenous brain-derived neurotrophic factor (BDNF) supplementation prevents tight junction (TJ) disruption and alleviates UC, suggesting that miR-151-5p targets BDNF to compromise TJ integrity (24). Similarly, miR-423-5p and miR-195-5p directly target claudin-5 and claudin-2, respectively, reducing TJ protein content (25, 26). Altered mucin expression further contributes to mucosal injury. In UC models, miR-429 is markedly downregulated, accompanied by reduced myristoylated alanine-rich C kinase substrate (MARCKS) levels. MARCKS plays a central role in the translocation of mucin-containing vesicles; its suppression by miR-429 leads to reduced secretion of MUC2 and MUC4, impairing the protective mucus layer and facilitating bacterial translocation. Besides, miR-429 modulates MARCKS-dependent secretion of mucins such as MUC2 and MUC4, influencing mucus layer integrity and permeability (27, 28). Collectively, miRNAs regulate UC progression by targeting apoptosis-related proteins, TJ components, and mucin secretion via defined signaling pathways. Such miRNA-mediated disruptions exacerbate mucosal permeability and accelerate disease progression, underscoring miRNA regulation of barrier integrity as a promising therapeutic target in UC.

### 2.2 miRNAs in innate immunity in UC

#### 2.2.1 Macrophages

Macrophages, highly phagocytic cells central to innate immunity, are critical in UC pathogenesis. In the colonic lamina propria, mucosal macrophages are significantly expanded and activated in UC patients compared to healthy individuals, underscoring their involvement in disease initiation and progression (29). Primarily derived from circulating monocytes, intestinal macrophages undergo local differentiation into classically activated (M1) or alternatively activated (M2) subsets, guided by microenvironmental signals (30). M1 macrophages, polarized by Th1-type cytokines and stimulated by IFN-γ, LPS, and TNF-α, produce pro-inflammatory mediators such as IL-1β, IL-12, IL-23, and TNF-α, and express inducible nitric oxide synthase (iNOS), thereby enhancing microbial clearance, recruiting inflammatory cells, and amplifying Th1/Th17 responses (31). Conversely, M2 macrophages, induced by Th2 cytokines (IL-4, IL-10, IL-13, TGF-β), release anti-inflammatory mediators (IL-4, IL-10, TGF-β) and chemokines (CCL17, CCL22, CCL24), which suppress Th1-mediated inflammation, promote Th2 immune polarization, and support epithelial restitution. In DSS-induced UC, miR-223 deficiency disrupts CX3CR1^hi^ macrophage differentiation and enhances their pro-inflammatory phenotype by relieving repression of CCAAT/enhancer-binding protein β (C/EBPβ) via its 3′UTR. C/EBPβ overexpression rescues these defects, highlighting miR-223 as a key regulator of macrophage development and intestinal homeostasis (32). The M1/M2 ratio reflects the inflammatory milieu, with M1 predominance aggravating inflammation and M2 predominance favoring repair, making polarization a therapeutic target (33). In a high-fat diet (HFD) and DSS-induced ulcerative colitis model, lipid vesicles generated under HFD conditions facilitate M1 macrophage polarization and exacerbate intestinal inflammation through transcription-dependent mechanisms. Therapeutic modulation of inflammatory signaling pathways attenuates macrophage infiltration, suppresses M1 polarization, promotes M2 phenotypic switch, and ultimately ameliorates disease severity (34).

#### 2.2.2 Dendritic cells

DCs are professional antigen-presenting cells capable of efficiently capturing, processing, and presenting antigens (35). Immature DCs function as potent inducers of T cell proliferation (36). Following maturation, they upregulate MHC class II and co-stimulatory molecules, thereby acquiring the capacity to prime naïve T cells and direct their differentiation toward Th1 or Th2 phenotypes (37). DC-derived IL-12, IL-16, and IL-23 promote Th1 responses, whereas Th2 cytokines (IL-4, IL-10) and regulatory T cells limit excessive Th1 activity, thereby reducing tissue injury (38). Given their pivotal role in sustaining immune homeostasis within genetically susceptible hosts, DC functionality is intimately associated with the pathogenesis of UC. Accumulating evidence highlights the contribution of specific microRNAs in modulating DC development, antigen presentation, and cytokine production (39). DC-specific intracellular adhesion molecule-3-grabbing nonintegrin (DC-SIGN, CD209), a C-type lectin receptor expressed on DC surfaces, interacts with various adhesion molecules and regulates immune responses (40). During monocyte-derived DC (moDC) differentiation, miR-181a promotes DC-SIGN expression and attenuates moDC responsiveness to both TLR ligands and CD40 engagement (41). Mechanistic studies demonstrate that miR-181a acts upstream of the ERK–MAPK pathway. Knockdown of miR-181a markedly reduces ERK phosphorylation upon GM-CSF and IL-4 stimulation, closely mirroring the effects of pharmacologic MEK1/2 inhibition by U0126 (42). Consistently, early blockade of ERK–MAPK signaling impairs DC-SIGN induction and skews differentiation toward a CD1a^+^CD14^+^DC-SIGN^-^ phenotype, reproducing the cellular signature observed in miR-181a–deficient conditions (42). These findings position miR-181a as a critical determinant of terminal moDC differentiation, acting through ERK–MAPK–dependent induction of DC-SIGN.

#### 2.2.3 Neutrophils

Neutrophils, the most abundant circulating immune cells, are essential for microbial clearance through phagocytosis, reactive oxygen species (ROS) generation, and neutrophil extracellular trap (NET) formation, processes that collectively help restore tissue homeostasis (43). However, excessive neutrophil activation in UC results in elevated ROS and protease release, which disrupt crypt architecture, amplify pro-inflammatory cytokine production, and further recruit neutrophils, thereby aggravating mucosal inflammation (44). Additionally, neutrophils also contribute to UC pathophysiology by facilitating lymphocyte migration and compromising epithelial barrier integrity. Recent investigations have revealed elevated expression of pro-inflammatory miR-23a and miR-155 in neutrophils infiltrating the colonic mucosa of UC patients. In clinical specimens and both *in vivo* and *in vitro* injury models, miR-155 was shown to promote DNA double-strand break accumulation by targeting nuclear lamina protein Lamin-B1 and suppressing RAD51-mediated homologous recombination repair, ultimately inducing acute colonic injury and genomic instability (45). Therapeutic inhibition of miR-23a and miR-155 in neutrophils reduces neutrophil infiltration, alleviates intestinal inflammation, and enhances mucosal repair in murine models of UC (45).

### 2.3 miRNAs in adaptive immunity in UC

#### 2.3.1 Th1/Th2 cells

Although the pathogenesis of ulcerative colitis (UC) remains incompletely understood, dysregulation of Th1 and Th2 cell responses, coupled with cytokine imbalance, constitutes a major contributor to the breakdown of immune homeostasis. Th1 cells secrete pro-inflammatory cytokines, including IFN-γ and TNF-α, that sustain local inflammation and aggravate tissue injury. Furthermore, disruption of the Th1/Th2 equilibrium is strongly associated with both the initiation and progression of UC (46, 47). In UC models, miRNAs have been identified as key modulators Th1/Th2 balance. For instance, upregulation of miR-19b promotes Th2 cell differentiation by increasing the expression of the transcriptional regulator Runx3, thereby influencing inflammatory processes (48). Similarly, miR-214-3p has been shown to influence Th1/Th2 polarization by targeting the anti-apoptotic protein B-cell lymphoma 2 (Bcl-2), leading to enhanced susceptibility of Th1 cells to apoptosis and a relative expansion of Th2 subsets. Moreover, miR-214-3p indirectly stabilizes the transcriptional activity of signal transducer and activator of transcription 6 (STAT6), a master regulator of Th2 differentiation, thereby promoting the expression of IL-4 and GATA3 while suppressing IFN-γ (49–51). Additionally, miR-21 contributes to UC pathogenesis by augmenting the synthesis and release of pro-inflammatory cytokines from colonic epithelial cells (49). Mechanistically, miR-21 targets and represses programmed cell death protein 4 (PDCD4), a known inhibitor of NF-κB and AP-1 transcriptional activity, thereby amplifying downstream IL-6 and TNF-α production and sustaining chronic mucosal inflammation (52).

#### 2.3.2 Th17/Treg cells

Aberrant activation of Th17 cells, combined with functional deficiency in Tregs, constitutes a fundamental immune imbalance in UC pathogenesis (53, 54). Th17 cells exhibit a pro-inflammatory phenotype, producing IL-17, IL-21, IL-22, and TNF-α, which recruit monocytes and neutrophils, thereby amplifying intestinal inflammation (55). In contrast, Treg cells mediate immunosuppressive functions largely through IL-10 secretion (56). Consequently, miRNA-mediated regulation of the Th17/Treg balance has emerged as a key area for mechanistic and therapeutic investigation in UC. The differentiation of Th17 cells is directed by the lineage-defining transcription factor retinoic acid–related orphan receptor C (RORC) (57). Pei et al. (58) demonstrated that miR-22 overexpression in CD4^+^ T cells upregulates RORC, driving Th17 differentiation and enhancing the production of IL-17A, IL-6, and TNF-α, without altering Th1/Th2-associated transcription factors (T-bet, GATA3) or cytokines (IFN-γ, IL-4). Through luciferase reporter assays, histone deacetylase 4 (HDAC4) was identified as a direct target of miR-22, with an inverse correlation in expression. Notably, HDAC4 also influences Foxp3 transcription and IL-10 expression in regulatory T cells (59). By targeting HDAC4, miR-22 may impair Treg lineage stability and reduce IL-10 production, thereby indirectly enhancing Th17 predominance and inflammatory amplification (60). miR-425 represents another critical regulatory molecule, demonstrating significantly elevated expression in UC patients. Yang et al. (10) reported that *in vivo* inhibition of miR-425 significantly reduces Th17 cell infiltration in colonic tissues. Mechanistic studies revealed that miR-425 directly targets the 3′UTR of forkhead box O1 (Foxo1)—a transcription factor that negatively regulates ulcerative colitis progression—leading to its suppression and consequently promoting Th17 differentiation and exacerbating intestinal inflammation. Foxo1 also serves as a key transcriptional activator of IL-10 in regulatory T cells and is essential for maintaining their immunosuppressive function (61). Thus, miR-425-mediated Foxo1 suppression may not only promote Th17 differentiation but also destabilize Treg phenotype and diminish IL-10 output, contributing to the skewed Th17/Treg balance in UC. These findings identify miR-425 or Foxo1 as potential therapeutic targets.

### 2.4 miRNAs in gut microbiota modulation in UC

**Figure 1:**
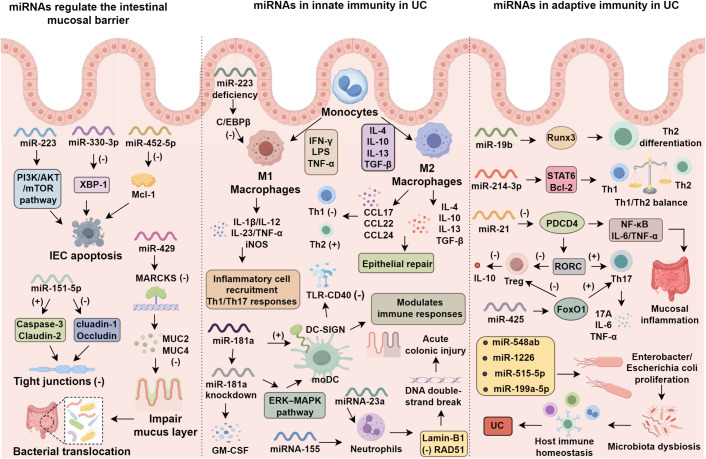
Roles of miRNAs in epithelial barrier integrity, innate immunity, and adaptive immune regulation in ulcerative colitis (UC).

UC is an autoimmune disease in which intestinal microbiota dysbiosis plays a critical role in perturbing host immune homeostasis (62). Recent studies have revealed that both endogenous and exogenous miRNAs can directly modulate the gut microbiota (63). Ji et al. (64) reported that several miRNAs—such as miR-548a, miR-1226, miR-515-5p, and miR-199a—are differentially expressed in fecal samples from UC patients and can directly target Enterobacter and Escherichia coli, thereby modulating their proliferation and contributing to UC progression. In a miR-21 knockout murine model, the number of operational taxonomic units (OTUs) within the gut microbiota was markedly diminished, and loss of miR-21 attenuated dysbiosis-driven susceptibility to DSS-induced colitis, indicating that miR-21 exacerbates colonic inflammation by shaping microbial communities (65). At the genus level, the abundance of *Salmonella*, *Anaerobiospirillum*, and *Oscillospira* decreased, while *Bifidobacterium* and *Aminobacterium* proportions increased, indicating that miR-21 can modulate microbiota composition by reducing *Bacteroides* abundance and promoting *Bifidobacterium* proliferation (65). Functionally, increased *Bifidobacterium* abundance has been shown to promote the differentiation of regulatory T cells and elevate IL-10 production, contributing to the restoration of mucosal immune tolerance and attenuation of intestinal inflammation (66). Conversely, depletion of *Oscillospira*, which is inversely associated with gut permeability, has been linked to enhanced bacterial translocation and activation of TLR4 signaling pathways in lamina propria macrophages, thereby amplifying NF-κB–mediated pro-inflammatory cytokine release (67). These findings underscore the immunoregulatory consequences of miRNA-induced microbiota shifts in UC. Beyond direct microbial targeting, miRNAs can indirectly influence the microbiota through modulation of immune cell activity, inflammatory mediators, and intestinal mucosal barrier function. Yu et al. (68) demonstrated that *Fusobacterium nucleatum* activates the TLR4/MYD88 signaling pathway to regulate the expression of miR-18a and miR-4802, which in turn suppress their autophagy-related gene targets—autophagy-associated gene 7 (ATG7) and Unc-51-like kinase 1 (ULK1). This inhibition impairs autophagic processes, thereby exacerbating UC (**Figure 1**). Moreover, bacterial extracellular vesicles (BEVs), released by both commensal and pathogenic bacteria, contain abundant RNA cargo, including small RNAs and host-interacting miRNA-like molecules, that can profoundly influence host immune responses partly through activation of Th1 and Th17 cell pathways (69).

## 3 miRNAs in UC clinical diagnosis, prognosis, and therapy

### 3.1 miRNAs as diagnostic, monitoring, and prognostic biomarkers in UC

Current UC diagnosis relies on invasive methods such as endoscopy and biopsy, which pose risks of perforation, hemorrhage, and mortality (70). miRNAs have emerged as promising non-invasive biomarkers for diagnosis, monitoring, and prognosis. Quantification of plasma miR-21 and miR-92a via RT-qPCR achieved around 90% sensitivity and specificity in differentiating UC from colorectal cancer, irritable bowel syndrome, and healthy controls (71). A two-miRNA panel (miR-598, miR-642) similarly enabled early UC detection (72). However, markers like miR-21 and miR-223 lack disease specificity, as both are elevated in Crohn’s disease, colorectal cancer, and even some IBS cases (71, 73–75). Thus, their diagnostic use should be contextualized alongside more UC-selective indicators. For disease monitoring, serum miR-223 demonstrates a stronger correlation with the Mayo endoscopic score (MES) than conventional biomarkers such as ESR or CRP (76, 77). A combined signature of miR-3615, miR-4792, and serum albumin accurately predicts a 90% one-year risk of treatment escalation (78). Furthermore, glucocorticoid-resistant UC patients consistently exhibit downregulation of miR-16-2-3p, miR-30e-3p, miR-32-5p, miR-642a-5p, miR-150-5p, and miR-224-5p (79), while a separate nine-miRNA signature effectively discriminates non-responders to biologic therapies (80). Collectively, miR-21, miR-92a, miR-598, miR-642, and miR-223 exhibit high diagnostic potential; miR-223 offers superior disease activity tracking, while other miRNAs predict prognosis and therapy responsiveness, underscoring their clinical utility in UC.

### 3.2 miRNA-targeted therapeutics in UC

Beyond the diagnostic value, miRNAs represent promising therapeutic agents for UC, especially in biologic resistance (81). Current approaches employ miRNA antagonists, chemically modified antisense oligonucleotides that inhibit target miRNAs, and mimics, which are synthetic double-stranded RNA oligonucleotides that restore or enhance miRNA activity. Among these, the miR-124–inducing agent ABX464 has shown particular clinical promise. This small molecule selectively upregulates miR-124 in human peripheral blood mononuclear cells and colonic macrophages, where it suppresses pro-inflammatory cytokines by targeting IL-6/STAT3 signaling. Although direct regulation of epithelial cells by miR-124 in UC remains unconfirmed (81, 82), preclinical DSS-induced colitis models demonstrated dose-dependent anti-inflammatory effects of ABX464 (83), primarily through miR-124 mediated downregulation of CCL2. CCL2 inhibition attenuates monocyte recruitment and promotes M2-like macrophage polarization, which facilitates mucosal healing via IL-10 and TGF-β secretion (84, 85). In a phase IIa trial (NCT03093259), ABX464 reduced adverse events compared to placebo and yielded superior endoscopic and clinical outcomes. Clinical remission and response rates were increased for placebo, and endoscopic improvement reached 50% (15). Patients with moderate-to-severe UC exhibited sustained improvements in modified Mayo scores, clinical response, remission, and endoscopic outcomes (86). Collectively, miRNA antagonists and mimics constitute a promising therapeutic class for UC, with ABX464 demonstrating robust efficacy and tolerability (**Table 1**).

**Table 1:** Representative miRNAs in ulcerative colitis: molecular targets, pathways, and clinical relevance.

miRNA	Validated target(s)	Affected pathway(s)	Functional role in UC	Clinical relevance	Ref
miR-223	C/EBPβ, PI3K/AKT/mTOR	Macrophage polarization, epithelial apoptosis	Regulates M1/M2 balance, promotes mucosal healing	Diagnostic (correlates with Mayo score); Therapeutic target for immune modulation	(22, 32–34)
miR-155	Lamin-B1, RAD51	DNA damage, NET formation	Enhances neutrophil activation, ROS, and inflammation	Therapeutic (targeted inhibition mitigates inflammation)	(45)
miR-21	PDCD4, gut microbial regulators	Th1/Th2 imbalance, microbiota composition	Sustains chronic mucosal inflammation	Diagnostic (circulating marker); Therapeutic target (knockout model)	(52, 65)
miR-22	HDAC4	RORC-mediated Th17 differentiation	Promotes IL-17, TNF-α, IL-6 production	Therapeutic (Th17 axis targeting in T cells)	(58–60)
miR-19b	Runx3	Th1/Th2 axis	Facilitates Th2 differentiation, modulates inflammation	Therapeutic (potential immune modulator)	(48)
miR-214-3p	Bcl-2	STAT6/Th1-Th2 equilibrium	Influences CD4^+^ T cell balance and cytokine secretion	Mechanistic insight; possible target	(49–51)
miR-425	FoxO1	Th17/Treg imbalance	Suppresses FoxO1 to promote Th17 cell differentiation	Therapeutic (inhibition reduces colonic infiltration)	(10, 61)

C/EBPβ, CCAAT/enhancer-binding protein beta; NET, neutrophil extracellular trap; STAT, signal transducer and activator of transcription; RORC, retinoic acid–related orphan receptor C; HDAC4, histone deacetylase 4; FoxO1, forkhead box protein O1; Ref., reference.

## 4 Conclusion

MicroRNAs (miRNAs) represent a convergent regulatory hub that orchestrates intestinal epithelial integrity, immune equilibrium, and microbial composition in ulcerative colitis (UC). Through their control over apoptosis, tight junction proteins, mucin secretion, and immune cell polarization, miRNAs bridge molecular events between epithelial injury and immune dysregulation. They also shape the gut microbial ecosystem, influencing host–microbe interactions that sustain or disrupt mucosal tolerance. This integrative role underscores miRNAs as both mechanistic drivers and dynamic indicators of UC pathogenesis, providing molecular insight into disease initiation, progression, and relapse.

Clinically, miRNAs have transcended their mechanistic importance to emerge as promising biomarkers and therapeutic agents. Circulating miRNAs offer non-invasive diagnostic and prognostic value, while therapeutic modulation, via mimics or inhibitors, shows encouraging efficacy, as evidenced by agents such as ABX464. Despite challenges including delivery efficiency, off-target effects, and pharmacokinetic stability, the translation of miRNA-targeted interventions represents a paradigm shift beyond conventional biologics. Future research integrating multi-omics profiling and targeted delivery systems will be pivotal to harness miRNAs for precision medicine, advancing the next generation of UC diagnostics and therapeutics.
